# Process evaluation of the Sophia Step Study- a primary care based three-armed randomized controlled trial using self-monitoring of steps with and without counseling in prediabetes and type 2 diabetes

**DOI:** 10.1186/s12889-021-11222-9

**Published:** 2021-06-22

**Authors:** Jenny Rossen, Maria Hagströmer, Agneta Yngve, Kerstin Brismar, Barbara Ainsworth, Unn-Britt Johansson

**Affiliations:** 1 Department of Health Promoting Science, Sophiahemmet University, Lindstedsvägen 8, Box 5605, 114 86 Stockholm, Sweden; 2 Division of Physiotherapy, Department of Neurobiology, Care Sciences and Society, Karolinska Institutet, Stockholm, Sweden; 3 Academic Primary Care Center, Region Stockholm, Stockholm, Sweden; 4 Department of Food Studies, Nutrition and Dietetics, Uppsala University, Uppsala, Sweden; 5 Department of Molecular Medicine and Surgery, Karolinska Institutet, Stockholm, Sweden; 6 Rolf Luft Research Center for Diabetes and Endocrinology, Karolinska University Hospital, Stockholm, Sweden; 7 School of Kinesiology, Shanghai University of Sport, Shanghai, China; 8 College of Health Solutions, Arizona State University, Phoenix, AZ USA; 9 Department of Clinical Science and Education, Södersjukhuset, Karolinska Institutet, Stockholm, Sweden

**Keywords:** Feasibility, Implementation, Pedometers, Physical activity, Prediabetes, Primary care, Self-monitoring, Type 2 diabetes

## Abstract

**Background:**

Describing implementation features of an intervention is required to compare interventions and to inform policy and best practice. The aim of this study was to conduct a process evaluation of the first 12 months of the Sophia Step Study: a primary care based RCT evaluating a multicomponent (self-monitoring of daily steps plus counseling) and a single component (self-monitoring of steps only) physical activity intervention to standard care on cardiometabolic health.

**Methods:**

The evaluation was guided by the Medical Research Council Guidance for complex interventions. To describe the implementation communication with the health professionals implementing the interventions, attendance records and tracking of days with self-monitored pedometer-determined steps were used. Change in physical activity behaviour was measured at baseline, 6 and 12 months as daily steps by accelerometry.

**Results:**

During April 2013 to January 2018 188 participants were randomized and intervened directly after inclusion. Response rate was 49% and drop out was 10%. A majority, 78%, had type 2 diabetes and 22% were diagnosed with prediabetes. Mean [Standard deviation (SD)] body mass index was 30.4 (4.4) kg/m^2^ and steps per day was 6566 (3086). The interventions were delivered as intended with minor deviation from the protocol and dose received was satisfying for both the multicomponent and single component group. The mean [95% Confidence Interval (CI)] change in daily steps from baseline to 6 months was 941(227, 1655) steps/day for the multicomponent intervention group, 990 (145, 1836) step/day for the single component group and − 506 (− 1118, 107) for the control group. The mean (95% CI) change in daily steps from baseline to 12 months was 31(− 507, 570) steps/day for the multicomponent intervention group, 144 (− 566, 853) step/day for the single component group and − 890 (− 1485, − 294) for the control group. There was a large individual variation in daily steps at baseline as well as in step change in all three groups.

**Conclusions:**

Applying self-monitoring of steps is a feasible method to implement as support for physical activity in the primary care setting both with and without counseling support.

**Trial registration:**

ClinicalTrials.gov, NCT02374788. Registered 2 March 2015.

**Supplementary Information:**

The online version contains supplementary material available at 10.1186/s12889-021-11222-9.

## Background

Type 2 diabetes and prediabetes are major global health concerns. The global prevalence rates for type 2 diabetes are expecting to escalate from 463 million people (year 2019) to 700 million (year 2045). The prevalence for impaired glucose tolerance (prediabetes) is 374 million (year 2019) [1]. It is well-established that physical activity improves metabolic control [2, 3] and cardiometabolic risk factors [4] in populations with prediabetes and type 2 diabetes. Diabetes care givers are encouraged to give advice on physical activity and such advice may be combined with recommendation on using a pedometer or a comparable self-monitoring device [5, 6]. Pedometers have shown positive effects on increased physical activity in short term in populations with type 2 diabetes [4, 7–10] and are recommended for use to increase motivation to be physically active [11, 12]. Yet, it is unclear whether self-monitoring alone, or in combination with counseling is the most feasible and effective alternative [9, 10, 13]. Comparison of available studies is difficult due to heterogeneous intervention setups and the limited reporting of context and implementation of interventions [14–16].

Sophia Step Study was a three-armed randomized controlled (RCT) trial evaluating pedometers and a digital tool for self-monitoring of steps with and without counseling by health care professionals on cardiometabolic health (primary outcome is HbA1c) [17]. The intervention duration was 2 years. The first year had a focus on changing behavior and included more intense support and the second year had a focus on maintenance of behavior and included follow-up at 18 months. In behavioral interventions of long duration many factors may influence both implementation and outcome [18]. To contribute to the scarce knowledgebase on implementation features of physical activity interventions in diabetes care a process evaluation of the Sophia Step Study was undertaken.

By describing context, implementation and behavior change outcomes, the intervention is made transparent and comparable, and variation in the outcomes of the intervention may be better understood [19]. In addition, making implementation factors explicit enables reproduction and informs policy on best practice [18, 19]. The effects of the RCT will be published in a separate paper.

## Methods

### Aim

The aim of this study was to undertake a process evaluation to describe the implementation and context as well as daily step behavior of the first 12 months of the Sophia Step Study.

### Study design

**Fig. 1:**
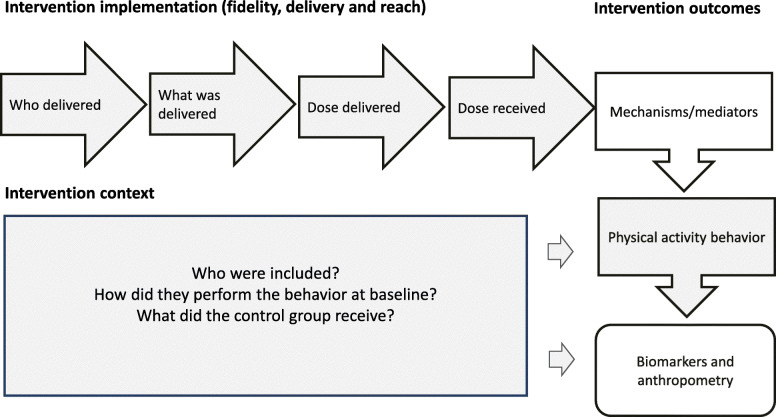
Framework for the process evaluation of Sophia Step Study. The shaded areas are described in this study

A process evaluation design adopted from the Medical Research Council Guidance (MRC) on context and implementation of complex interventions was applied [19]. Context refers to external and contextual factors that may affect the implementation and effects of an intervention. Implementation includes fidelity (whether the intervention was delivered as intended), delivery (what was delivered in practice), adaptations (if adjustments were made) and reach (whether and how the intended population group came in contact with the intervention) [19]. Figure 1 depicts a framework for the process evaluation plan. Mechanisms for behavior change have been explored and reported previously [20].

### Context

Participants were recruited by their diabetes specialist nurse at an urban primary care centre and an insurance clinic in central Stockholm, and at a primary care centre in a rural area in southern Sweden between April 2013 and January 2018. Approximately 385 persons were assessed for eligibility by their diabetes specialist nurse or general practitioner and invited to participate, consecutively or by a mailed invitation.

Criteria for inclusion were 40–80 years; having prediabetes (HbA_1c_ > 39- < 47 mmol/mol and/or fasting glucose > 5.6 mmol/l) or diagnosed with type 2 diabetes with a duration of > 1 year and ability to communicate in Swedish. Exclusion criteria were myocardial infarction in the past 6 months; serum creatinine > 140 mmol/l; diabetic foot ulcer or risk of ulcer (severe peripheral neuropathy); on insulin since the last 6 months; co-morbidity prohibiting physical activity; repeated hypoglycemia or severe hypoglycemia in the past 12 months; having no access to internet, or being very physically active according to the Stanford Brief Activity Survey [21]. Being very physically active comprised: having a work that include hard physical labor most of the day, or engaged in exercise, or other vigorous intensity activities, for 30 min at least 3 times per week. A power calculation was made and is described in the study protocol [17]. Sample size was determined to 56 participants per group, assuming to detect a difference of > 0.6 mmol/mol in HbA1c at 12 months between group A and B and between group A and C. At the baseline assessment the invited participants were asked to fill out a questionnaire including demographics, emailed or paper based as they preferred. At follow-ups roughly the same questions were asked. Specific details on included questionnaires are published in a study protocol [17]. The participants were randomized into one of three groups (allocation ratio 1:1:1) using sealed envelopes. Dropout rate at 12 months was 10%. Standard care included meeting a diabetes specialist nurse and a physician once a year, or more often if the blood glucose levels were high or unstable. All participants were offered study assessments at baseline and month 2, 3, 4, 6, 9 and 12 including feedback on health outcomes, which should be regarded as a brief intervention per se.

### Interventions

The aims of the interventions in Sophia Step Study were to support individuals in increasing their physical activity levels and subsequently improve glucose metabolism control (primary outcome is HbA_1c_) and to reduce cardiovascular risk. The full programme duration was 2 years, the first year had a focus on behavior change and the second year applied behavior change techniques for maintaining change. In this study the first 12 months were explored. The program details and theoretical framework are described in the study protocol [17]. Participants allocated to the two intervention arms (a multicomponent intervention, A, or a single component intervention, B) were offered pedometers (Yamax Digiwalker SW 200: Yamax Corporation, Tokyo, Japan) and a website for self-monitoring of steps (steg.se) by their diabetes specialist nurse. Group A participants were, in addition to pedometers and the website, offered nine group consultations and five individual face-to-face consultations during the first intervention year [22]. The individual consultations were based on motivational interviewing and physical activity on prescription; a health-care provider prescription to emphasize health-enhancing physical activity [23]. Three diabetes specialist nurses were involved in the intervention. The nurses were conventionally trained in motivational interviewing, in the applied behavior change theories and in the method to prescribe physical activity. The group sessions added more intense support and interaction to the intervention, as well as social support, role modelling and behavioural capacity. The group sessions were led by project staff (the urban centres) and the diabetes specialist nurse (rural centre). Group C served as a control group with standard care.

### Implementation

The project staff communicated continuously with the nurses to inquire about fidelity to the intervention protocol, possible challenges arising, and adaptations made during the study and collected attendance records protocols and notes.

The nurses were asked to evaluate the quality of each of the motivational interviewing consultations on a scale 1–10, to log their reflections on the consultations and to report if physical activity on prescription had been applied and followed up. Reflections from the group consultations for group A were logged briefly. Dose received (participant adherence to the intervention components) was drawn from attendance records and data from the website for step registration.

### Physical activity behavior outcome

Daily steps were measured over 7 days at baseline, 6 and 12 months with ActiGraph GT1M accelerometer (ActiGraph, Pensacola, FL). The accelerometers and instructions were handed out during study assessments and the participants came by the primary care center to leave them or posted them back by prepaid envelope. Procedures for data collection and processing have been published elsewhere [24]. Non-wear time was set at 90 min with consecutive zero counts, with allowance for 2 min intervals of nonzero counts [25]. Participants providing data of ≥10 h per day for at least 3 days were included in the analyses [26]. Steps were also dichotomized to: reach ≥5000 steps or not and reach ≥7000 steps or not. Taking less than 5000 steps/day has been proposed as having a sedentary lifestyle and taking more than 7000 steps/day as having an active lifestyle [27].

### Statistical analyses

The Statistical Package for Social Science, SPSS (IBM Corp. Released 2019. IBM SPSS Statistics for Windows, Version 26.0. Armonk, NY: IBM Corp) was used for the statistical analyses. The data was examined for normality, outliers and missing data. Within group differences on changes after 6 and 12 months were analysed using paired sample t-tests. Multiplicity was not controlled for and *P*-values should therefore not be interpreted.

## Results

### Context

**Fig. 2:**
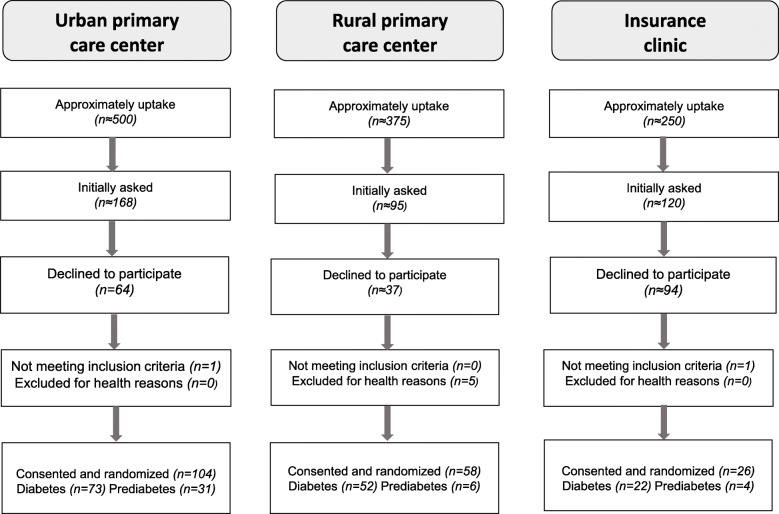
Study flow diagram of enrollment for Sophia Step Study

By November 2018, 188 individuals fulfilled inclusion and exclusion criteria, agreed to participate, and were randomized. Response rate of eligible and invited individuals was 49%. Most named reasons for declining participation were already active; time constraints; long distance to health care center; have no interest or because of health reasons. Figure 2. provides the number of participants invited, declined, eligible and consented and randomized at the respective primary care center.

Table 1 describes baseline characteristics of the randomized participants per allocated group.

**Table 1:** Baseline characteristics by intervention group

	Group A	Group B	Group C	Total
	*n* = 64	*n* = 59	*n* = 65	*n* = 188
Age, years	64.2 (6.8)	65.1 (7.3)	63.1 (8.7)	64.1 (7.7)
Female, %	44%	41%	37%	40%
Prediabetes, %	20%	19%	26%	22%
Diabetes duration^a^, years	9.4 (7.1)	7.8 (5.1)	7.2 (4.8)	8.2 (6.0)
Daily smoker	7%	10%	6%	7%
University education	49%	43%	61%	51%
Living with partner	75%	71%	70%	72%
Body Mass Index, kg/m^2^	30.3 (4.1)	28.6 (4.5)	30.3 (4.7)	30.1 (4.4)
Accelerometer wear time, min/day^b^	838.8 (92.3)	835.6 (59.6)	839.4 (65.0)	838.0 (74.1)
Steps/day^b^	6532 (2892)	6605 (3293)	6561 (3122)	6566 (3086)
> 5000 steps/day^b^	68%	67%	60%	65%
> 7000 steps/day^b^	41%	42%	39%	41%
Vegetables, daily servings	1.4 (0.8)	1.5 (1.1)	1.7 (1.1)	1.5 (1.0)
Percentage whole wheat bread of consumed bread	80 (28)	82 (31)	80 (28)	81 (29)
Cooking fat quality, mostly butter	33%	27%	21%	27%

The table presents mean (standard deviation) or proportion (%). The number of participants vary with 1–2 for some variables due to missing data

^a^Only participants diagnosed with type 2 diabetes

^b^Baseline accelerometry data were available for 163 participants

For the total sample, most were diagnosed with type 2 diabetes and body mass index (BMI) levels classified 46% of the participants as obese (≥30 kg/m^2^). Fewer than 1/3 were classified as having a sedentary lifestyle with an average of 6566 steps per day. About half had a university education, a majority were men and less than 10% were current smokers. Characteristics of participants from each primary care center are provided in Additional file 1 to illustrate the characteristics of participants from the different contexts.

### Implementation

#### Fidelity, delivery, and reach

The intervention components were delivered as planned with minor adjustments. Physical activity on prescription was applied occasionally as the diabetes specialist nurses felt it did not add any value above the use of self-monitoring of steps in combination with motivational interviewing. The ratings and logs of motivational interviewing were missing for more than half of the sample and evaluation of the quality of the MI sessions was not possible. Two of the diabetes specialist nurses experienced the consultations as difficult to rate, but all three were in overall satisfied with the consultations. The group sessions followed the planned program, except that one session was cancelled for one group during summer. Some issues during the intervention were reported: low attendance at group sessions (due to work, illness or other obligations); the individual consultations occasionally discussed health matters rather than support for physical activity; technical problems with the pedometers; lost passwords; and temporary problems accessing the website. The technical problems were solved instantly, and these issues were not considered affecting the delivery of the intervention, but to be regarded as natural deviations in clinical practice. No adaptations were made to the study protocol during the intervention, although the participants could use another step counter such as a Fitbit or smart phone if they felt the pedometer was not reliable.

**Fig. 3:**
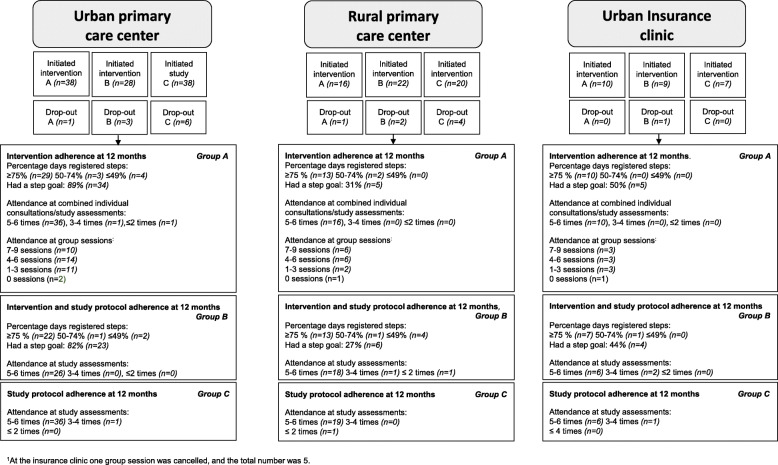
Intervention flow diagram showing number of participants adhering to the respective intervention component, by study center

The flow chart in Fig. 3 shows dose received (participants’ adherence to the intervention components) by respective primary care center. Mean percentage of days with registered steps on the website over 12 months was 88% for both group A and B. In group A 73% and in group B 65% had an individual step goal. No adverse health events due to participation were reported during the first 12 months intervention.

### Physical activity behavior

Mean differences in steps from baseline to 6- and 12 months for each allocated group are listed in Table 2. Daily steps increased to a similar extent between baseline and 6 months for Group A and B. Between baseline and 12 months group A did not change and Group B increased mean daily steps marginally. Steps declined for Group C at each measurement period. Changes in minutes of accelerometer wear-time at each measurement period was negligible for all groups.

**Table 2:** Mean differences in daily steps and accelerometer wear time between baseline and 6 months and baseline and 12 months for each intervention group

	Multicomponent group A Mean change (95% CI) (n)	Single component group B Mean change (95% CI) (n)	Control Group C Mean change (95% CI) (n)
Daily steps baseline- 6 months	941 (227, 1655) (*n* = 40)	990 (145, 1836) (*n* = 43)	− 506 (− 1118, 107) (*n* = 37)
Wear time (min) baseline- 6 months	12.6 (− 19.5, 44.8) (*n* = 40)	11.0 (−4.8, 26.8) (*n* = 43)	14.8 (−11.0, 40.6) (*n* = 37)
Daily steps baseline- 12 months	31 (− 507, 570) (*n* = 46)	144 (−566, 853) (*n* = 39)	−890 (− 1485, − 294) (*n* = 39)
Wear time (min) baseline- 12 months	− 10.1 (− 29.3, 9.0) (*n* = 46)	−0.7 (− 21.8, 20.1) (*n* = 39)	8.6 (− 14.2, 31.4) (*n* = 39)

**Fig. 4:**
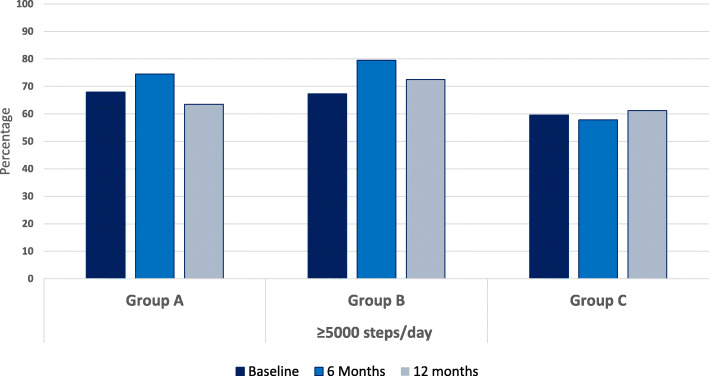
Percentage in each intervention group reaching 5000 steps or more at baseline and after 6- and 12-months intervention

**Fig. 5:**
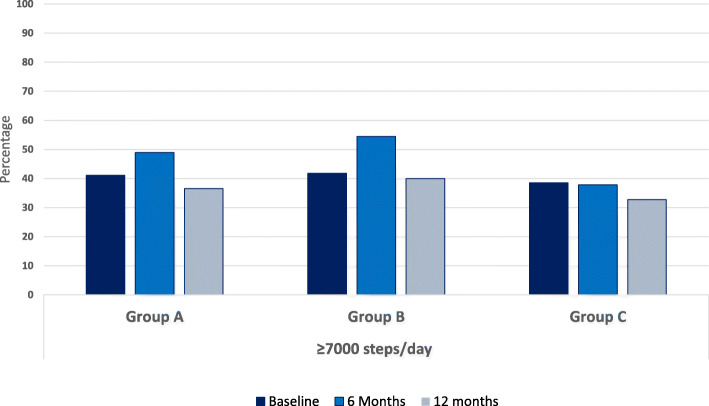
Percentage in each intervention group reaching 7000 steps or more at baseline and after 6- and 12-months intervention

Figures 4 and 5 display the percentage of participants in each allocated group reaching 5000 respectively 7000 steps per day at baseline, 6- and 12 months.

At least 60% of participants in all groups maintained at least 5000 steps per day and nearly 40% reached 7000 steps per day at the baseline, 6- and 12-month assessment periods. Both group A and B showed increases in number of participants reaching the 5000 and 7000 steps per day thresholds at the 6-months, however these increases declined at 12 months. The proportion of Group C participants taking at least 5000 steps per day was consistent across the intervention period, while the proportion of group C participants reaching 7000 steps per day declined at 12 months.

**Fig. 6:**
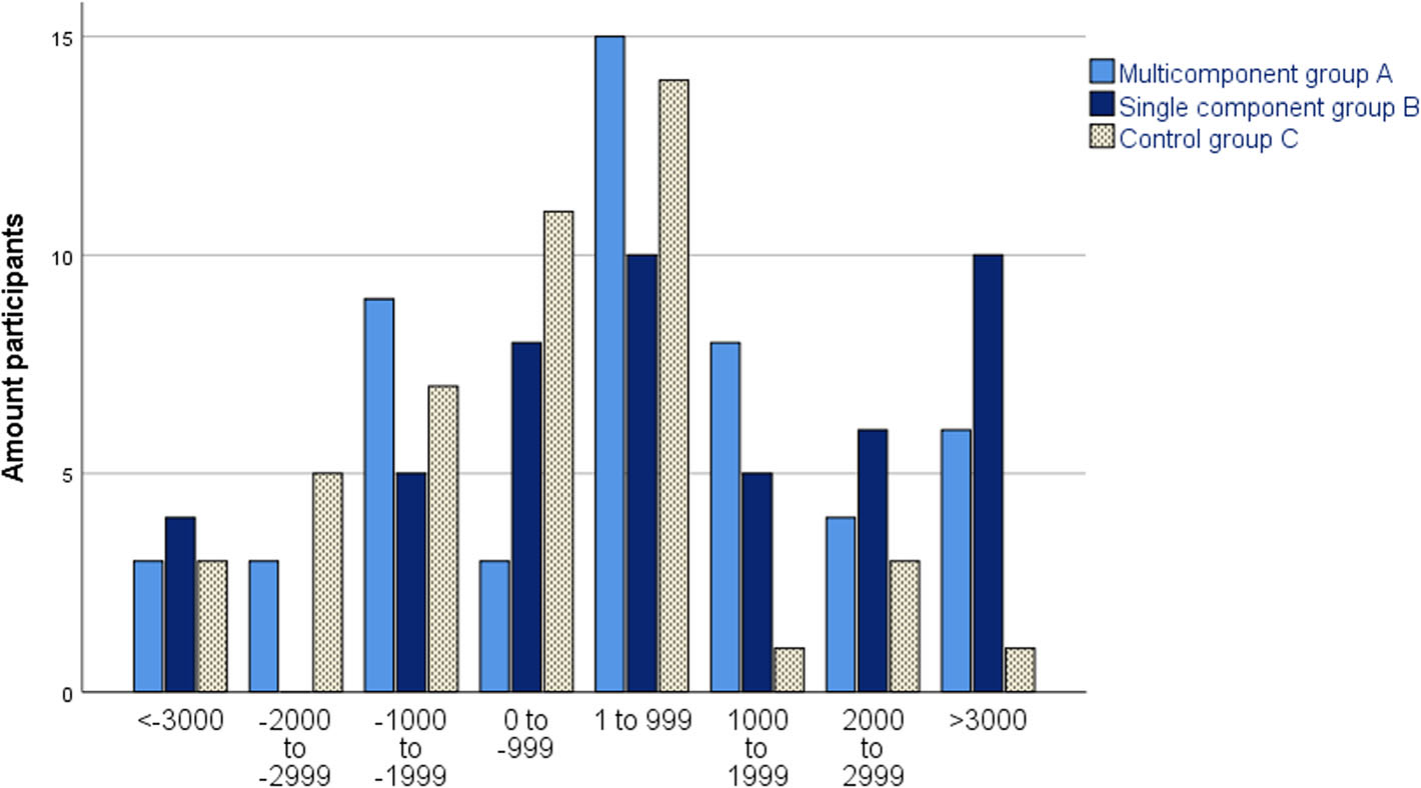
Distribution of change in steps from baseline to 6 months for respective allocated group

**Fig. 7:**
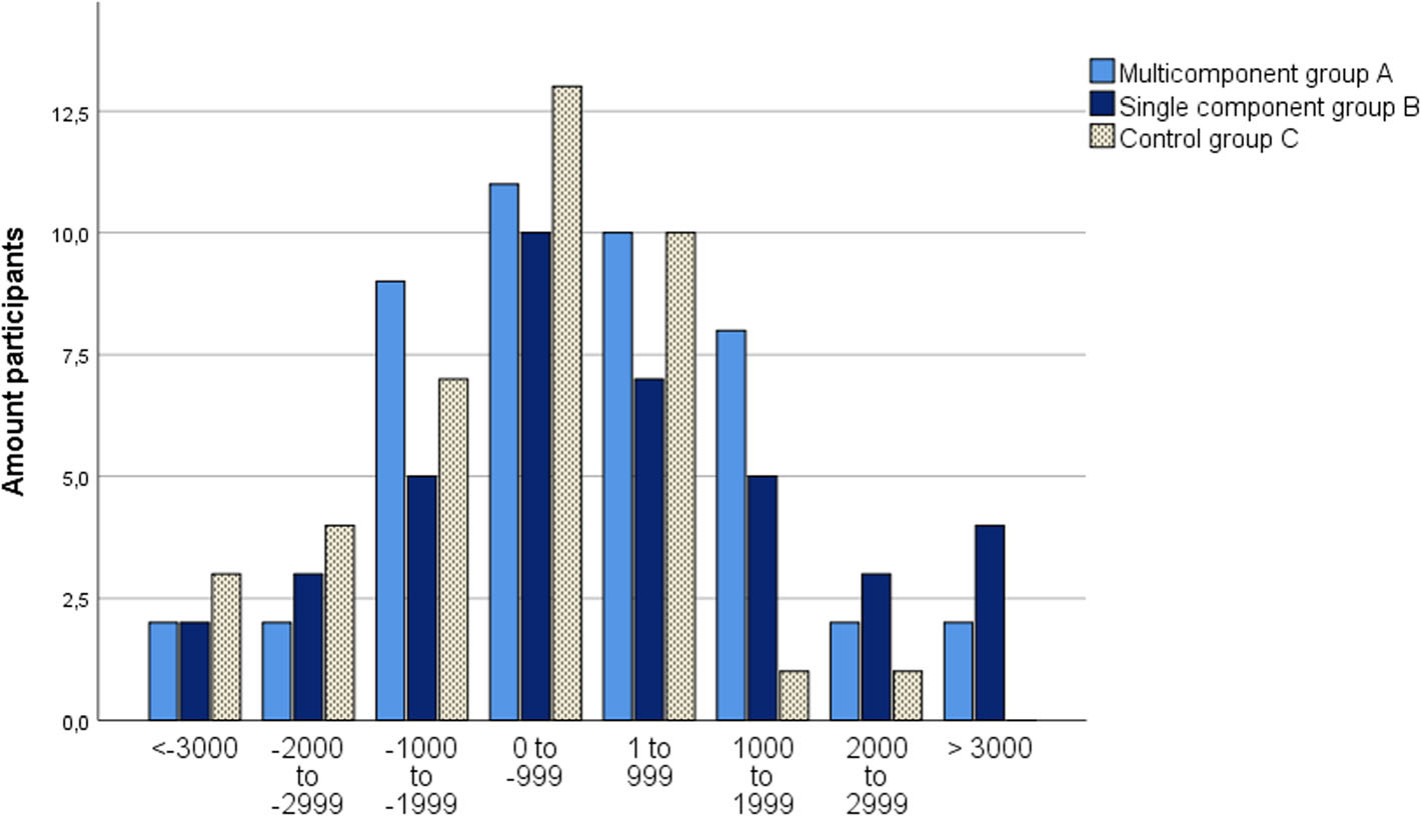
Distribution of change in steps from baseline to 12 months for respective allocated group

The distribution of step change for respective allocated intervention group is shown in intervals of 1000 steps from baseline to 6 months in Fig. 6 and from baseline to 12 months in Fig. 7. There was a spread in the level of step change among the participants in each group and the distribution of changes in the 1000 steps appeared normally distributed for each group at both assessment periods. At 6 months, most participants increased from 1 to 999 steps per day, with an exception of Group B where 10 participants increased by 3000 steps per day or more. At 12 months, a decreased by 0 to − 999 steps per day was the most common change.

## Discussion

This study demonstrates that self-monitoring of steps both with and without counseling are feasible methods to implement in the primary care setting to increase and maintain daily steps in individuals with prediabetes and type 2 diabetes. The response rate (49%) is comparable to similar interventions and is probably a realistic number to reach with a behavior change program [28–30]. Both genders were equally represented in this study, and the proportion of individuals with prediabetes corresponded well to the proportions listed at the health care centers. A majority of the participants were recruited from urban centers, and every other participant had university education, which are factors that reduces the external validity of the feasibility. Many of the participants in this study were rather active at baseline (mean steps 6566 steps/day) which is in congruence with previous studies in type 2 diabetes [29–31] and pointing to the fact that behavioral interventions reach individuals with high disease engagement or already healthy lifestyles, rather than those in most need.

Both interventions were delivered with high fidelity to the study protocol, with the exceptions of the intervention component physical activity on prescription not implemented as expected and the quality of the consultations not being evaluated. It is important to notice that without solving technical issues that emerged with the pedometers and the website instantly, the adherence rate had possibly been lower. The dropout rate during the first 12 months was 10% and dose received can be considered as high for both intervention groups. Similar findings with high adherence to pedometer interventions have been shown in earlier studies [28, 32, 33], suggesting that individuals who enroll are highly motivated and prepared to adhere to physical activity interventions. A larger proportion of participants at the urban primary care center, than at the other two centers, had a step goal, both in the group with counseling and the group that did not receive counseling. Having a step goal has been shown to be of importance for success in previous pedometer interventions and it is of importance to further explore this behavior change technique [7, 34].

The results of this study add to the body of evidence that self-monitoring of steps is an effective method in increasing physical activity up to 6 months [7–10]. The increase in physical activity seems to be difficult to maintain on a group level over 12 months. Similar change in daily steps after 12 months intervention was observed in the Walking Away from Type 2 Diabetes trial [35]. A large part (41%) of the sample in this study already reached recommended levels of 7000 steps/day at baseline and to increase PA further in this group is challenging. For these individuals maintaining the physical activity levels over the years is a reasonable approach. This study shows individual variation in the number of daily steps at baseline, but also in change of daily steps in all three groups. Considering the large variation in daily steps at baseline as well as response to the interventions, further research should focus on methods to tailor support more precisely based on circumstance around the individual.

The hypothesis of Sophia Step Study is that the multi-component intervention (group A) has superior effects compared to the single component intervention and that the effects are better maintained in group A over the course of 2 years. After 6 months, participants in group B, (who were not offered counseling), seemed to increase their average daily steps to the same extent, or even slightly more than group A. After 12 months both intervention groups returned to their respective baseline mean daily step level. Similar results were demonstrated in the PACE-UP trial, with the group receiving pedometers by mail increasing physical activity to the same extent as the group receiving pedometers and nurse support [36]. In comparison, in the HEALD trial, the group receiving pedometers without any counseling (similar to our group B) increased daily steps to a lower extent than the group receiving counseling [31]. Meta-analyses provide conflicting results regarding the efficacy of counseling as a complement to self-monitoring [9, 10]. The specific content of the intervention components may influence both the implementation and the outcomes of an intervention. In Sophia Step Study group B participants met the diabetes specialist nurse shortly during the study assessments and this may have influenced the participants’ motivation. In a previously published qualitative interview study participants’ experiences of Sophia Step Study were explored. The findings of the qualitative study revealed that the study assessments included for all study participants were appreciated as encouraging as they gave feedback on health outcomes [20]. This may have influenced the motivation for self-management for all three groups.

Among the strengths of this study is the reporting of process features and making the implementation transparent, which will assist in interpreting the final study outcomes and allows replication and comparison of the intervention [19]. Another strength of the study is the use of objective measures of physical activity from an accelerometer rather than using the self-reported steps from the website.

There are aspects of fidelity that could have been further explored and reported, e.g. training of nurses, coding of motivational interviewing and the quality of group consultations. A further limitation of this process evaluation is the limited range of the included participants regarding their demographic profile. The trial has not been evaluated on a low socioeconomic population, nor on a group with limited skills in the Swedish language. It is likely that adjustments would be needed if self-monitoring of steps were applied on other population groups.

## Conclusions

Applying self-monitoring of steps is a feasible method to implement as support for physical activity in the primary care setting both with and without counseling support. While physical activity levels increased after 6 months, maintenance of physical activity is a more realistic expectation in the long term.

## Supplementary Information

**Additional file 1: Supplementary Table.** Characteristics per primary care center.

## Data Availability

The datasets generated and/or analyzed during the current study are not publicly available since data can be traced back to the study participants. According to Swedish and EU data legislation this means that access can only be available from the corresponding author on reasonable request. Any sharing of data will be regulated via a data transfer and use agreement with the recipient.

## Abbreviations

BMI: Body mass index
MRC: Medical Research Council Guidance
RCT: Randomized controlled trial
