# Supplementary material for: Process evaluation of the Sophia Step Study- a primary care based three-armed randomized controlled trial using self-monitoring of steps with and without counseling in prediabetes and type 2 diabetes
Source: BMC Public Health. 2021 Jun 22;21:1191. doi: 10.1186/s12889-021-11222-9 (PMC8220758; doi:10.1186/s12889-021-11222-9)
Supplement: Supplementary file 1 — Additional file 1: Supplementary Table. Characteristics per primary care center. [file 12889_2021_11222_MOESM1_ESM.docx]

Supplementary table. Baseline characteristics by primary care center.

|  | Urban primary care center | | Rural primary care center | Urban insurance clinic | Total |
| --- | --- | --- | --- | --- | --- |
|  | n =104 | n = 58 | | n =26 | n = 188 |
| Age, years | 64.6 (7.2) | 65.0 (8.3) | | 60.1 (7.2) | 64.1 (7.7) |
| Female, % | 54% | 19% | | 35% | 40% |
| Prediabetes, % | 30% | 10% | | 15% | 22% |
| Diabetes duration^1^, years | 8.6 (5.5) | 8.8 (6.8) | | 5.3 (3.2) | 8.2 (6.0) |
| Daily smoker | 4% | 15% | | 8% | 7% |
| University education | 59% | 27% | | 58% | 51% |
| Living with partner | 66% | 81% | | 81% | 72% |
| Body Mass Index, kg/m^2^ | 30.0 (4.6) | | 30.7 (4.2) | (4.2) | 30.1 (4.4) |
| Accelerometer wear time, min/day^2^ | 833.0 (83.6) | | 840.5 (83.6) | 852.6 (66.9) | 838.0 (74.1) |
| Steps/day^2^ | 6999.3 (2920.4) | | 5164.0 (2851.0) | 8109.0  (3078.4) | 6566  (3086) |
| > 5000 steps/day^2^ | 73% | | 43% | 83% | 65% |
| > 7000 steps/day^2^ | 45% | | 25% | 58% | 41% |
| Vegetables, daily servings | 1.6 (1.0) | | 1.3 (1.0) | 1.5 (1.0) | 1.5 (1.0) |
| Percentage whole wheat bread of consumed bread | 80% (29%) | | 83% (24%) | 80% (32%) | 81 (29) |
| Cooking fat quality, mostly butter | 25% | | 25% | 39% | 27% |

Table presents mean (standard deviation) or proportion (%). The number of participants vary with 1-2 for some variables due to missing data.
^1^ Only participants diagnosed with type 2 diabetes
^2^ Baseline accelerometry data were available for 163 participants
